# Alterations in circadian entrainment precede the onset of depression-like behavior that does not respond to fluoxetine

**DOI:** 10.1038/tp.2015.94

**Published:** 2015-07-14

**Authors:** S Spulber, M Conti, C DuPont, M Raciti, R Bose, N Onishchenko, S Ceccatelli

**Affiliations:** 1 Department of Neuroscience, Karolinska Institutet, Stockholm, Sweden

## Abstract

Growing evidence links adverse prenatal conditions to mood disorders. We investigated the long-term behavioral alterations induced by prenatal exposure to excess glucocorticoids (dexamethasone—DEX). At 12 months, but not earlier, DEX-exposed mice displayed depression-like behavior and impaired hippocampal neurogenesis, not reversible by the antidepressant fluoxetine (FLX). Concomitantly, we observed arrhythmic glucocorticoid secretion and absent circadian oscillations in hippocampal clock gene expression. Analysis of spontaneous activity showed progressive alterations in circadian entrainment preceding depression. Circadian oscillations in clock gene expression (measured by means of quantitative PCR) were also attenuated in skin fibroblasts before the appearance of depression. Interestingly, circadian entrainment is not altered in a model of depression (induced by methylmercury prenatal exposure) that responds to FLX. Altogether, our results suggest that alterations in circadian entrainment of spontaneous activity, and possibly clock gene expression in fibroblasts, may predict the onset of depression and the response to FLX in patients.

## Introduction

Major depressive disorder is a major cause of disability, and has high economical and personal costs.^1^ Apparently easy to recognize, major depressive disorder is a clinical entity with multiple endophenotypes and multifactorial etiology.^2, 3^ Environmental factors, such as childhood abuse/neglect or exposure to food contaminants, can cause epigenetic changes in early life, which may lead to mood disorders in adults, as shown in humans and rodents.^4, 5, 6^ Recent epidemiological studies point to a correlation between intrauterine growth retardation and depression.^7^ Similarly, animal models have shown that prenatal stress, glucocorticoid (GC) exposure and inhibition of 11β-hydroxysteroid dehydrogenase type 2 (the placental enzyme that inactivates maternal GC) reduce birth body weight and increase the occurrence of pathological conditions in adults, including dysregulation of the hypothalamic–pituitary–adrenal axis with subsequent alterations in circadian rhythms and anxiety-related behaviors.^8^

Neurotransmitters imbalance is believed to have a major role in the etiology of depression (monoamine hypothesis; see Nutt^9^). According to the neurogenic theory, impaired adult hippocampal neurogenesis has a major role in the onset of depression, and antidepressant treatment leads to recovery by restoring neurogenesis.^10, 11, 12, 13^ We have previously shown that the synthetic GC dexamethasone (DEX) and the environmental contaminant methylmercury (MeHg) share a number of effects on the differentiation potential of human and rodent embryonic neural stem cells, and both induce persistent changes related to senescence.^14, 15^ In addition, developmental exposure to MeHg induces long-lasting depression-like behavior associated with impaired hippocampal neurogenesis that are reversed by antidepressant treatment with fluoxetine (FLX).^4, 16^

In this study we investigated the possible occurrence of depression-like behavior induced by prenatal exposure to DEX. We found that DEX-exposed mice aged 12 months (mo), but not younger, displayed depression-like behavior and impaired hippocampal neurogenesis, which did not respond to antidepressant treatment with FLX. Given the delayed onset of the phenotype, we searched for earlier/progressive alterations that would precede the onset of depression, and possibly predict the response to treatment. Depression in humans is most often accompanied by a history of disturbed biological rhythms.^17, 18^ Therefore, we analyzed the homecage spontaneous behavior in DEX-exposed mice before and after the onset of depression-like behavior, and identified progressive alterations in circadian entrainment that appeared long before depression. We next sought to confirm the alteration in circadian entrainment in a relevant peripheral system, minimally invasive, that can be readily translated into the clinical setting, namely skin fibroblast cultures (see Nagoshi *et al.*^19^, Welsh *et al.*^20^, Pagani *et al.*^21^, Bamne *et al.*^22^ and Lippert *et al.*^23^). In agreement with the circadian entrainment alterations in spontaneous activity, fibroblasts isolated from DEX-exposed mice displayed lower amplitude of oscillations in clock gene expression. We then asked whether the alterations in circadian entrainment can be associated with the response to antidepressant treatment. To this end, we analyzed the circadian entrainment of spontaneous activity in mice exposed to MeHg, in which depression is reversed by FLX.^4^ Altogether, our data suggest that alterations in circadian entrainment precede the onset of depression-like behavior, and possibly predict the response to FLX. Of particular relevance is the correspondence between the blunted oscillations in clock gene expression in the brain and skin fibroblasts, both occurring before the onset of depression-like behavior. These findings suggest that alterations in circadian entrainment provide a potential biomarker for identifying subjects at risk of developing depression as well as for predicting the response to antidepressant treatment, and point to fibroblasts as a promising read-out system. Our findings can be readily implemented in a clinical setting, as circadian entrainment can be investigated in human subjects by means of wrist actigraphy^24^ and by analyzing the expression of clock genes in skin fibroblasts.^19, 20, 21, 22, 23^

## Materials and methods

### Animals and treatments

All experiments were performed in agreement with the European and Swedish national regulation following approval by the local Animal Ethics Committee (Stockholms Norra djurförsöksetiska nämnd).

Timed-pregnant C57Bl/6 mice (*N*=34 per treatment) (Charles River, SCANBUR Research, Sollentuna, Sweden) were injected subcutaneously with 0.05 mg kg^−1^ per day DEX (Sigma-Aldrich, Stockholm, Sweden) from gestational day (GD) 14 (the day the postcoital plug was noted was considered GD0) until delivery (recorded as postnatal day (PND) 0). This dose induces a moderate fetal growth retardation without affecting litter size, gestational length or maternal behavior.^25^ Control mice were delivered by sham-treated females, that is, they were handled the same way, at the same time of the day as the exposed dams, and were injected with an equivalent volume of vehicle. The litters were culled to four pups per litter at PND3 and the pups were weighed at PND 3, 7, 14 and 21.

The exposure to MeHg has been described elsewhere.^16^ Briefly, pregnant C57Bl/6 dams (*N*=6 per treatment) received MeHg (CH_3_HgOH) at the dose of 0.5 mg kg^−1^ per day via drinking water from GD7 till day 7 after delivery. Control dams received tap water.

At weaning (PND21), the mice were implanted subcutaneously with sterile radio frequency identification tags (Trovan Unique 100A, Trovan, Douglas, Isle of Man, UK) under brief isoflurane anesthesia. The transponders allow unambiguous identification of animals and are also used for monitoring the activity in a homecage environment. After implantation, the pups were redistributed to new cages so that each cage would house a maximum of five mice originating from different litters, and the distribution was maintained throughout the study. The mice were kept in an animal facility under 12:12-h light–dark (LD) cycle (light intensity 50 lx; light on at 0600 hours) at constant temperature (22±1 °C) and humidity (50±5%).

All experiments involving circadian rhythms were performed in an isolated room (22±1 °C; 50±5% relative humidity), with a 12:12-h LD cycle (light intensity 200 lx; light on at 0600 hours). The circadian zeitgeber ('time giver') time (ZT) 0 corresponds to the onset of the light phase. The interaction with human experimenters was limited to changing the cage and replenishing food and water, which occurred at random times throughout the experiment.

### Behavioral testing

The mice were first screened in a battery of behavior tests, including open field, and a social recognition test (see Supplementary Materials and Methods).

Adult male mice aged 7 weeks, 3 mo or 12 mo were tested for depression-like behavior in the forced swim tests, as described earlier.^16^ Briefly, the animals were individually placed in glass cylinders (24 cm height, 12 cm diameter) filled with water (23.5 °C) to a depth of 16 cm. The animals were exposed to a 15-min pre-test followed by a 6 min test 24 h later. Test sessions were videotaped and analyzed offline by one investigator who was blind to the treatment and exposure conditions. Immobility was defined as passive floating for at least 2 s. After documenting depression-like behavior at the age of 12 mo, the animals were treated with FLX (a selective serotonin reuptake inhibitor antidepressant) dissolved in drinking water (80 mg l^−1^) for 21 days before repeating the test. This dosage did not reduce water intake and resulted in FLX plasma levels within therapeutic range in humans.^4, 26^ In addition to forced swim tests, we tested depression-like behavior in a tail-suspension test, as described in the Supplementary Materials and Methods.

### Analysis of hippocampal neurogenesis and GR expression

The subgranular zone of the hippocampal dentate gyrus (DG) is one of the brain regions that retain the neurogenic potential in adult animals.^27^ To investigate neurogenesis, we estimated the progenitor proliferation and the maturation of newly generated neurons in the DG by immunohistochemical methods (see Supplementary Materials and Methods). Progenitor proliferation was assessed by counting EdU-positive cells in the subgranular zone (defined as a two-cell-diameter layer subjacent to the granule cell layer of the DG) after pulse-labeling by systemic administration of EdU (50 mg kg^−1^ per day intraperitoneally at ZT12 for 7 days). Maturation of newly generated neurons was assessed by counting doublecortin (DCX)-positive neuroblasts throughout the granule cell layer of the DG. The cell counting was performed in a stereological design using vertical sections. Equally spaced series of sagittal sections (20 μm thick; 200 μm between consecutive slices) were collected starting from the first occurrence of the hippocampal structure, until the dorsal hippocampal commissure (lateral 3.5–0 mm in stereotaxic coordinates^28^). The total number of cells was estimated by multiplying the number of counted cells with the inverse sampling fraction.

The expression of the glucocorticoid receptor (GR) was assessed by measuring the fluorescence intensity in the DG. The intensity of the positive signal was estimated in the granule cell layer (manually delineated), and the background intensity was evaluated in the molecular and polymorph layers of the DG.

### Corticosterone metabolites in feces

We investigated the diurnal rhythm of GC secretion at 12 mo by collecting spontaneous fecal boli between ZT12 and ZT12-14 (that is, immediately after the transition between the light and dark phases). The feces were collected in sterile Eppendorf tubes and stored at −80 °C until further processing. Samples from each mouse (*N*=8–10 per group) were collected on two occasions (7-day interval between samplings). The concentration of corticosterone metabolites in dry fecal extracts was measured by enzyme immunoassay according to the manufacturer's instructions (DetectX, Arbor Assays, Ann Arbor, MA, USA).

### Analysis of clock gene expression in the hippocampus

We investigated the expression of clock genes in hippocampi harvested from male mice 3 and 12 mo (*N*=4 per group) maintained in a 12:12-h LD cycle for at least 7 days before sampling. The mice (two mice per cage) were killed at ZT 3 and ZT12 (corresponding to the peak and trough in messenger RNA (mRNA) expression of core clock genes involved in the main feedback loop, such as *Bmal1* and *Per/Cry*; see also Harbour *et al.*^29^) by an overdose of anesthetic (sodium pentobarbital, 150 mg kg^−1^). The blood was removed by transcardial perfusion with ice-cold buffered saline. The hippocampus was quickly dissected on ice and stored at −80 °C until processing. The relative expression of clock genes was assessed by quantitative real-time PCR with *Gapdh* as a housekeeping gene (see also Supplementary Materials and Methods). The clock genes (*Bmal1*, *Per1* and *Rev-Erb α*) were selected for analysis based on their involvement in the core feedback loops of molecular clock, as well as on their documented regulation of neurogenesis.^30, 31, 32^

### Analysis of spontaneous activity

We recorded the spontaneous activity of mice aged 1, 3, 5 and 12 mo (*N*=7–8 per group) using the TraffiCage system (NewBehavior, Zürich, Switzerland), as described elsewhere.^33^ Briefly, the system consists of an array of antennas placed under the cage with group-housed, freely moving mice. The antennas read the radio frequency identification tags and provide an approximate location of each animal with a time resolution of 20 ms. A 'visit' was defined as the time interval during which an animal is detected constantly by the same antenna, and was used as activity count. The time series of visits are exported as ASCII files and analyzed using custom algorithm implementations in Matlab R2013b (The MathWorks, Natick, MA, USA).

To minimize the effects of novelty and possible interference from circadian rhythm entrainment, we derived the baseline measurements based on three LD cycles after an acclimation period of at least three LD cycles. The mice were then exposed to constant darkness (DD; free-running period) for 2 weeks. Spontaneous activity in free-running conditions was analyzed over the last 72 h of recording in DD. Circadian re-entrainment was induced by resuming the LD cycle. The effects of forced resynchronization were analyzed during the first 3 days of circadian re-entrainment. Each recording session included two cages monitored simultaneously, one control and one test cage, placed randomly on either TraffiCage plates.

We analyzed spontaneous activity by detrended fluctuation analysis. The method is based on linear regression analysis of the residual variance of the time series against the timescale used for detrending on double-logarithmic plots. The correlation coefficient of a linear regression in double-logarithmic plot translates into a scaling exponent, and describes the long-term autocorrelation patterns embedded in the time series.^34^ Random fluctuations yield a scaling exponent around 0.5, and values close to 1 suggest strong underlying regularity; complex time series with fractal-like patterned irregularity yield values between 0.5 and 1.^34^ The scaling exponent in young, healthy rodents and humans is around 0.8,^35, 36^ and the loss of patterned irregularity has been suggested to be a hallmark of disease.^37^

The analysis of circadian rhythmicity consisted of rhythmometry by means of cosinor analysis.^38, 39^ The period of spontaneous activity was estimated as the highest peak in the *χ*^2^-periodogram^40^ between 20 and 25 h with a 5-min resolution.

### Analysis of active phase in relation to the LD cycle

The activity counts were binned in 5-min non-overlapping epochs, and then smoothed with a weighted average using a sliding Gauss window (4 h width). The epochs with activity above the individual's average were considered 'active epochs'. The active phase was defined as a sequence of active epochs contiguous or separated by gaps no larger than 1 h. The onset and the offset of the active phase were defined as the ZT corresponding to the beginning and the end of the active phase, respectively. The duration of the active phase was calculated as the time span between the onset and the offset of the active phase during one LD cycle. For steady entrainment conditions, the analysis of active phase is based on three consecutive LD cycles.

### Code availability

The Matlab routines developed for TraffiCage data analysis are available upon request.

### Primary fibroblast cultures from adult mice

Tissue samples (~0.25 cm^2^) were harvested from the ear of adult (6 mo) control and Dex-exposed mice under terminal anesthesia. The tissue was rinsed in Hank's balanced salt solution (Life Technologies Europe, Stockholm, Sweden), then minced with sterile razor blade into Collagenase (Type XI-S) (Sigma-Aldrich) (30 min at 37 °C). After digestion, 3 ml of Dulbecco's modified Eagle's medium (DMEM; Life Technologies Europe) supplemented with 10% fetal bovine serum and 1% penicillin/streptomycin (Life Technologies Europe) was added to a 6-cm plate and the samples were incubated at 37 °C for at least 6 days. After passaging (0.05% Trypsin-EDTA; Invitrogen, Life Technologies Europe), the cells were plated in 12 multi-well plates in MEF medium (DMEM medium+10% fetal bovine serum+1% pen/strep) at a density of at least 50k cm^−^^2^. After 24 h, the expression of clock genes was synchronized by exposing the fibroblasts to 1μm Dex. The cells were collected between 6 and 36 h after synchronization. The relative expression of *Bmal1* was assessed by quantitative PCR with *Gapdh* as the housekeeping gene (see also Supplementary Materials and Methods). Circadian oscillations in clock gene expression were analyzed by means of cosinor rhythmometry.^38, 39^

### Statistical analyses

All statistical analyses were performed in Statistica version 12 (Statsoft Scandinavia, Uppsala, Sweden). Unless otherwise specified, we used simple, factorial or mixed (repeated measures between-group) design analysis of variance models followed by unequal *N* HSD *post hoc* test or contrast analysis. The results are shown as average and s.e.m. The number of independent samples in each group is indicated in the figure legend. The statistical power of all analyses is higher than 0.8.

## Results

### Outcomes of prenatal exposure to DEX

The exposure to DEX from GD14 until delivery induced a mild but consistent decrease in intrauterine growth rate (Supplementary Figure 1A). After delivery, the body weight was lower in both male and female DEX-exposed mice, as previously shown in rats.^25, 41^ The difference was consistent until weaning (PND21), but disappeared soon thereafter (no significant difference at PND28; Supplementary Figure 1B). The behavioral outcomes of prenatal exposure to DEX, including hyperactivity in the open field and impaired social behavior in the social recognition test, were only present in the male offspring (Supplementary Figure 2). The sex differences were in agreement with previous studies,^42, 43^ and therefore we used only the male offspring for the following experiments.

### Mice exposed to DEX prenatally display depression-like behavior that is not reversed by FLX

**Figure 1:**
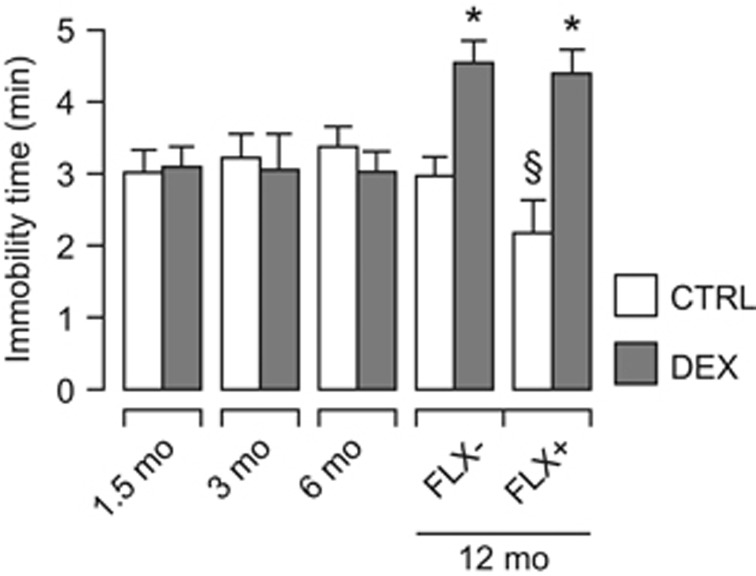
DEX-exposed mice show depression-like behavior that is not reversed by antidepressant treatment with FLX. Note that depression becomes manifest at 12 mo, but not earlier. Chronic treatment with FLX does not decrease immobility time in DEX-exposed mice, but only in controls. Factorial ANOVA followed by contrast analysis. *N*=6–10 per group per time point. *P<0.05 between-group; §*P*<0.05 within-group. ANOVA, analysis of variance; CTRL, control; DEX, dexamethasone; FLX, fluoxetine; mo, months.

We tested male offspring in the forced swim test at several ages, and found that DEX-exposed mice showed increased immobility time at 12 mo, but not earlier (Figure 1). The depression-like phenotype was confirmed in the tail-suspension test (Supplementary Figure 3). We next asked whether antidepressant treatment could reverse the behavioral phenotype. We selected FLX based on our previous experience^4^ and on the established effectiveness of selective serotonin re-uptake inhibitors in models of depression induced by prenatal stress.^44, 45^ Treatment with FLX for 21 days before repeating the forced swim test did not affect the immobility time in DEX-exposed mice, but decreased it only in controls (Figure 1).

### Prenatal exposure to DEX induces alterations in neurogenesis that are not reversed by FLX

**Figure 2:**
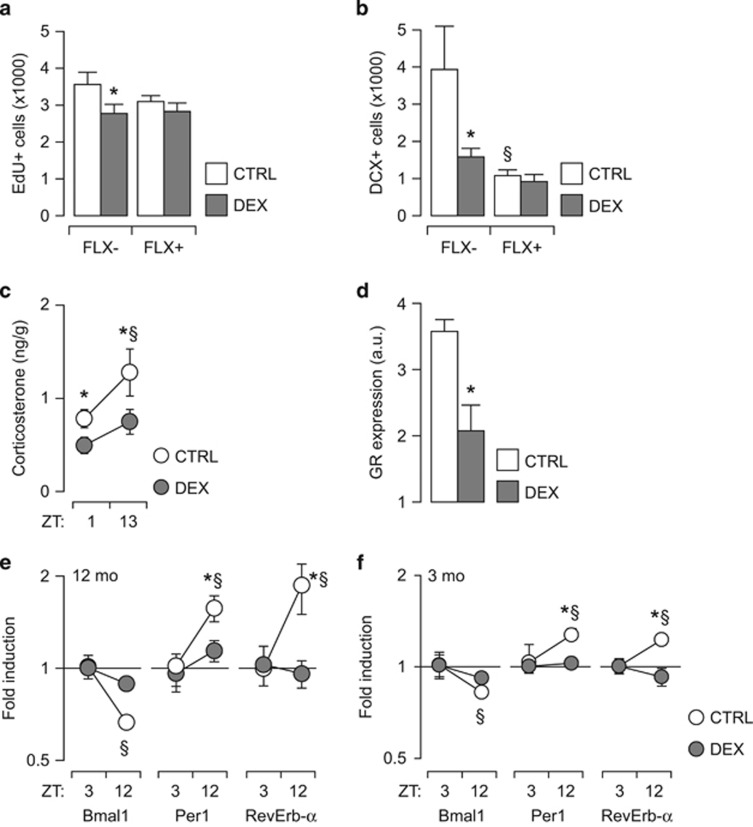
Long-lasting defects in hippocampal neurogenesis associated with depression. (**a**, **b**) Impaired hippocampal neurogenesis in DEX-exposed mice. Developmental exposure to DEX reduces both progenitor cell proliferation (EdU+ cell number) (**a**), and neuronal differentiation (DCX-positive cell number) (**b**). Chronic antidepressant treatment with FLX has no effect in DEX-exposed mice, but reduces neuronal differentiation in controls. (**c**) DEX-exposed mice have lower levels of corticosterone metabolites in the feces and display no circadian fluctuations. (**d**) GR expression in the DG is lower in DEX-exposed mice than in controls. (**e**, **f**) Expression of clock genes in the hippocampus at 12 (**e**) and 3 (**f**) mo. Circadian fluctuations in the hippocampal expression of *Bmal1*, *Per1* and *Rev-Erb α* are abolished in DEX-exposed mice. (**a**, **b**, **e**, **f**) Factorial ANOVA followed by contrast analysis. (**c**) Student's *t*-test. (**d**) Mixed-model ANOVA (repeated measures between-group design), followed by unequal *N* HSD *post hoc* test; *N*=6–8 per group. *P<0.05 between-group; §*P*<0.05 within-group. ANOVA, analysis of variance; DG, dentate gyrus; DCX, doublecortin; DEX, dexamethasone; FLX, fluoxetine; GR, glucocorticoid receptor; mo, months; ZT, zeitgeber time.

We investigated neurogenesis at 12 mo of age and observed that DEX-exposed mice had a lower number of EdU-positive cells in the subgranular zone (Figure 2a) and less DCX-positive cells in the granular layer of the DG (Figure 2b). FLX treatment did not have any significant effect on neurogenesis in DEX-exposed mice. However, in agreement with earlier reports,^46, 47, 48^ FLX decreased the number of DCX-positive cells in controls.

The antidepressant effect of FLX requires rhythmic GC secretion,^49^ therefore we investigated the diurnal rhythm of GC secretion by measuring the concentration of GC metabolites in feces. DEX-exposed mice exhibited lower levels of corticosterone metabolites that did not show significant diurnal oscillations (Figure 2c).

Hypothalamic–pituitary–adrenal axis activity regulation, including the circadian fluctuations in GC secretion, involves hippocampal GR in the feedback loop.^50, 51^ The analysis of GR expression in the hippocampus revealed that 12 mo-old DEX-exposed mice had a significantly lower GR signal intensity in DG (Figure 2d; see also Supplementary Figure 4), in agreement with earlier reports.^52, 53^

Neurogenesis timing and progression are regulated by clock genes, particularly by *Bmal1* and *Per1*,^30, 31^ and oscillations in circulating GC induce their expression in the hippocampus.^54, 55^ The quantitative PCR analysis of clock genes in the hippocampi showed that in 12-mo-old depressed DEX-exposed mice there were no diurnal oscillations in the expression of *Bmal1*, *Per1* and *Rev-Erb α* (Figure 2e). We next asked whether similar alterations in clock gene expression may occur before the onset of depression-like behavior, and analyzed hippocampi harvested from 3-mo-old mice. Notably, the diurnal oscillations in *Bmal1*, *Per1* and *Rev-Erb α* were abolished already at a young age in DEX-exposed mice (Figure 2f).

### Alterations in circadian entrainment precede depression-like behavior

**Figure 3:**
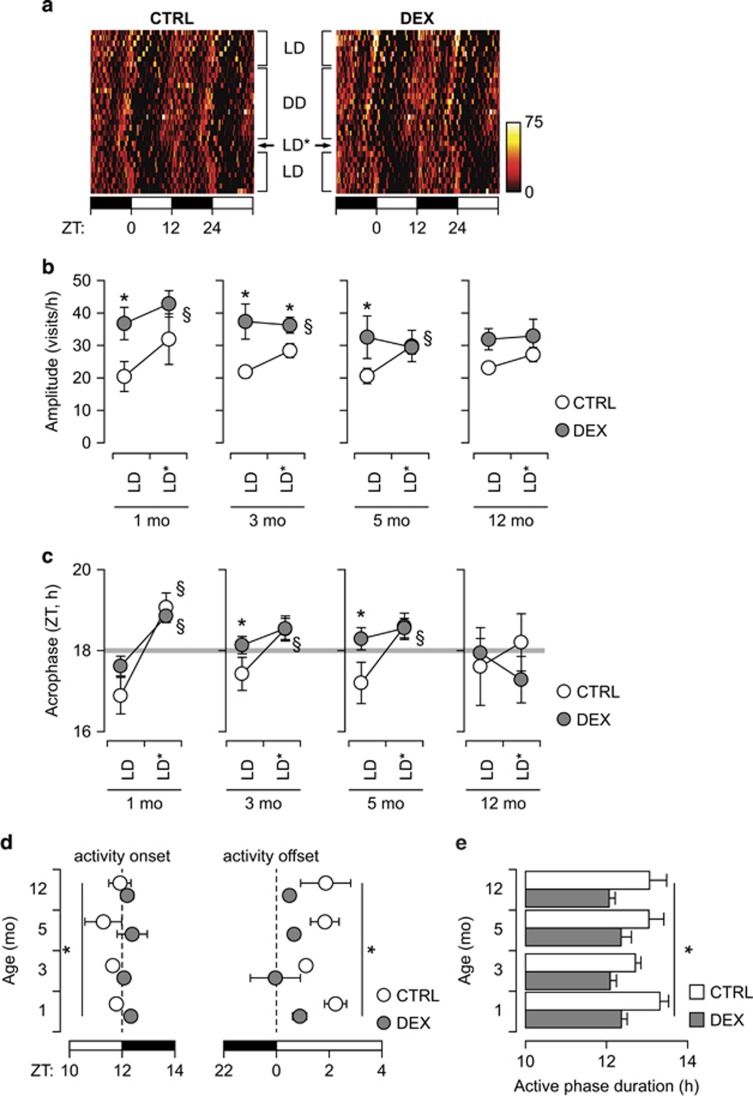
Alterations in circadian rhythmicity precede the onset of depression-like behavior. (**a**) Representative double-plotted heatmap in 3-mo CTRL and DEX-exposed (DEX) mice. Each point represents the cumulative number of visits recorded in 15-min time bins. The light and dark phases of the LD cycle are indicated by the black and white rectangles, respectively, at the bottom. LD—steady entrainment; LD*—forced synchronization; DD—constant darkness (free-running). (**b**) Amplitude of circadian rhythm measured by cosinor analysis during steady entrainment (LD) and after forced synchronization (LD*). The amplitude does not vary between baseline and forced synchronization in DEX-exposed mice aged 3 mo and older. (**c**) Delayed acrophase of spontaneous activity, and no difference between steady entrainment (LD) and forced synchronization (LD*) in DEX-exposed mice aged 3 mo and older. (**d**) Analysis of onset and offset of active phase in relation to the LD cycle. The active phase onset occurs earlier in controls than in DEX-exposed mice at all ages. Spontaneous activity is rapidly suppressed after the beginning of the light phase in DEX-exposed mice, whereas in controls the active phase ends later. The light and dark phases are depicted by the white and black rectangles, respectively, at the bottom of the graphs. (**e**) Quantification of the active phase duration. In DEX-exposed mice, the active phase is shorter than in controls, essentially equal to the duration of the dark phase of the LD cycle. (**a**–**c**) Mixed-model ANOVA (repeated measures between-group design), followed by unequal *N* HSD *post hoc* test; *N*=6–8 per group. *P<0.05 between-group; §*P*<0.05 within-group. (**d**, **e**) Factorial ANOVA followed by contrast analysis (*N*=6–8 per group); *P<0.05 between-group. ANOVA, analysis of variance; CTRL, control; DEX, dexamethasone; LD, light–dark cycle; mo, months; ZT, zeitgeber time.

Diurnal fluctuations in GC are also involved in regulating circadian entrainment of spontaneous activity, particularly in response to changes in the LD cycle.^56, 57, 58, 59^ Therefore, we analyzed the locomotor activity in the homecage in steady-state conditions (LD cycle or DD), as well as the response to circadian re-entrainment (forced synchronization; Figure 3a). Circadian entrainment allows the anticipation of regular events (for example, onset of dark/light phase). In spontaneous activity, this is illustrated by the acrophase occurring before ZT 18 (that is, the middle of the dark phase) in the context of steady entrainment with a 24-h circadian period. In contrast, forced synchronization transiently increases the amplitude of circadian oscillations and delays the acrophase to around ZT 18. DEX-exposed mice displayed larger amplitude than controls during steady entrainment already from 1 mo of age, and showed no significant difference between steady entrainment and forced synchronization (Figure 3b). The acrophase in DEX-exposed mice occurred close to ZT 18 at all ages tested, and was significantly delayed by forced synchronization only at 1 mo (Figure 3c). In contrast, the acrophase of spontaneous activity in control mice occurred consistently before ZT 18 under steady entrainment conditions, and was significantly delayed by forced resynchronization at 1, 3 and 5 mo (Figure 3c). This suggested that photic entrainment was stronger in DEX-exposed mice than in controls, particularly in steady entrainment conditions. Therefore, we next analyzed the onset and the offset of the active phase in relation to the LD cycle. We found that the onset of active phase virtually coincides with the beginning of the dark phase in DEX-exposed mice, but precedes the dark onset in controls (Figure 3d). In contrast, the offset of the active phase occurs very soon after the end of the dark period in DEX-exposed mice, whereas in controls it occurs consistently later than the end of the dark phase (Figure 3d). This led to DEX-exposed mice having a shorter active phase than controls at all ages tested (Figure 3e).

**Figure 4:**
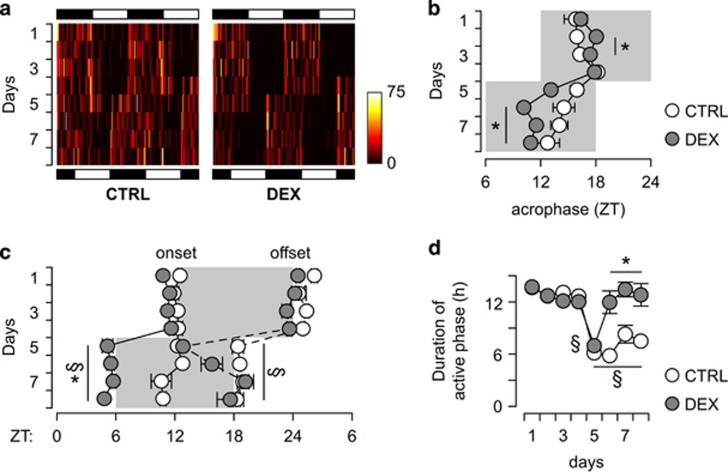
Changes in spontaneous activity in response to a 6-h phase advance in 6 mo-old mice. (**a**) Representative double-plotted heatmaps of spontaneous activity in 15-min bins. The dark and light phases in the beginning and at the end of the experiment are indicated by the black and white rectangles at the top and bottom of the figure, respectively. (**b**) Estimation of acrophase relative to the LD cycle (dark phase depicted as the shaded area). Note that DEX-exposed mice adapt faster to the phase advance, and the acrophase is immediately synchronized with the dark phase. (**c**) Detection of spontaneous activity onset and offset in relation to the LD cycle (dark phase depicted by the gray-shaded area). In DEX-exposed mice, the active phase follows the onset and the offset of the dark phase immediately after the phase shift, and is perfectly aligned after only two LD cycles. In contrast, the active phase onset gradually advances from the previously entrained rhythm in controls, and the active phase is terminated by the onset of the light phase. (**d**) In DEX-exposed mice, the duration of the active phase is constant around 12 h (with the exception of the first LD cycle after phase shift), whereas in controls the active phase is markedly shortened after the phase shift. (**b**, **c**) Mixed-model ANOVA (repeated measures between-group design), followed by unequal *N* HSD *post hoc* test; *P<0.05 between-group; §*P*<0.05 within-group. (**d**) Factorial ANOVA followed by unequal *N* HSD *post hoc* test; *N*=7–8 per group. *P<0.05 between-group. ANOVA, analysis of variance; CTRL, control; DEX, dexamethasone; LD, light–dark cycle; ZT, zeitgeber time.

To confirm this hypothesis, we analyzed the spontaneous activity in response to a 6-h phase advance at the age of 6 mo (Figure 4a) and observed that the acrophase is advanced by 6 h immediately after the phase advance in the LD cycle (Figure 4b). When we analyzed the onset and the offset of the active phase in relation to the LD cycle, we found that the onset of active phase coincides with the beginning of the dark phase immediately after the phase advance in the LD cycle. In contrast, the onset of activity is lagging behind the onset of the dark phase in control mice (Figure 4c). In addition, the offset of activity in DEX-exposed mice occurs earlier during the first two LD cycles after the phase advance, but later coincides with the offset of the dark phase (Figure 4c). In control mice, the onset of the light phase suppresses spontaneous activity immediately after the phase advance in the LD cycle (Figure 4c). This pattern of changes is reflected in the duration of the active phase, which is shortened only during the first LD cycle after the phase advance in DEX-exposed mice, whereas in controls it was persistently shorter after the phase advance. Altogether, these data suggest that the diurnal rhythms in spontaneous activity in DEX-exposed mice are more rigid in relation to the LD cycle, and that DEX-exposed mice entrain a circadian rhythm considerably faster than controls.

**Figure 5:**
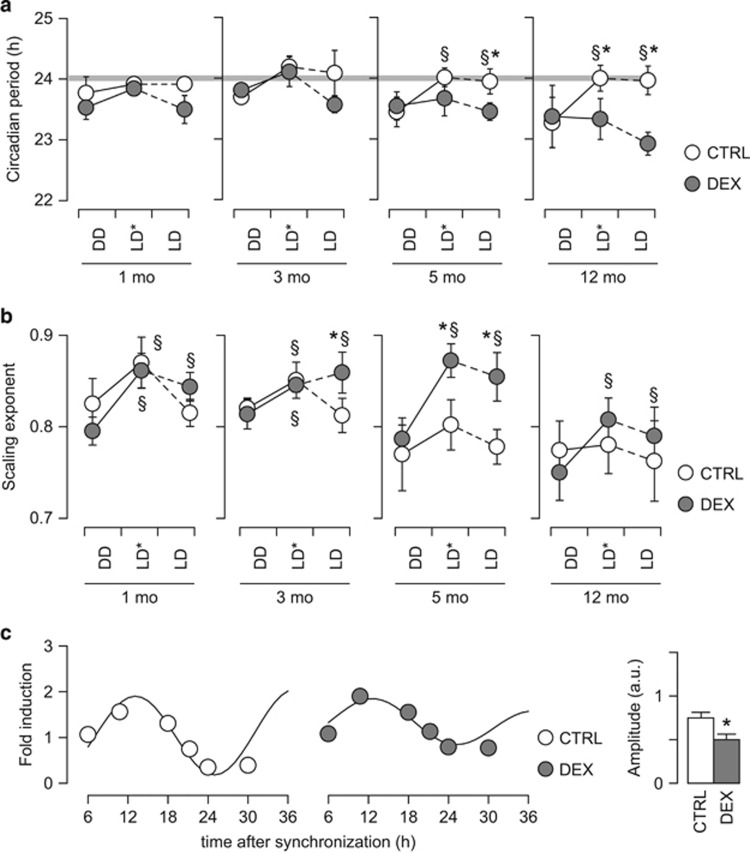
Alterations in circadian entrainment in DEX-exposed mice. (**a**) Internal period of spontaneous activity in the homecage. The free-running period is not different between DEX-exposed mice and controls. Forced synchronization (LD*) entrains a stable circadian period of 24 h in controls at all ages tested. Young DEX-exposed mice entrain a circadian rhythm during forced synchronization, but the circadian period during steady entrainment is shorter than in controls at the age of 5 mo. At 12 mo, circadian entrainment fails in DEX-exposed mice even during forced synchronization. (**b**) Scaling exponent for the spontaneous activity in the homecage. Note that during free-running (DD), DEX-exposed mice are not different from controls at any age tested. However, diurnal entrainment (both forced synchronization, LD*, and steady entrainment, LD) induces a consistent increase in the scaling exponent as compared with free-running periods (DD) in DEX-exposed mice. In controls, the scaling exponent during steady entrainment (LD) is not different from free-running conditions (DD). (**c**) Expression of *Bmal1* in cultured fibroblasts. At the age of 6 mo, fibroblasts isolated from DEX-exposed mice display attenuated oscillations in *Bmal1* mRNA expression. (**a**, **b**) Mixed-model ANOVA (repeated measures between-group design), followed by unequal *N* HSD *post hoc* test; *N*=6–8 per group. *P<0.05 between-group; §*P*<0.05 within-group; (**c**) *N*=3 per group; *P<0.05, Student's *t*-test. ANOVA, analysis of variance; CTRL, control; DEX, dexamethasone; LD, light–dark cycle; mo, months.

Circadian entrainment also implies that the synchronized oscillation is self-sustained, and has a period similar to that of the zeitgeber. Forced synchronization induced circadian re-entraining in both controls and DEX-exposed mice, and the circadian period was close to 24 h at young ages (Figure 5a). However, in young DEX-exposed animals during steady entrainment the circadian period displayed a tendency to deviate from 24 h, and the difference became significant in steady entrainment at the age of 5 mo (Figure 5a). Moreover, at 12 mo, circadian entrainment failed in DEX-exposed mice even during forced synchronization, and the circadian period did not vary between free-running, forced synchronization and steady entrainment (Figure 5a).

To investigate whether the alterations in circadian rhythmicity are due to central clock dysfunction, or solely due to diurnal entrainment, we evaluated the effects of photic entrainment in comparison with free-running conditions. In free-running conditions, intrinsic rhythmicity in spontaneous activity is controlled by the central clock located in the suprachiasmatic nucleus.^35, 36^ We found no difference between DEX-exposed mice and controls in either free-running period or scaling exponent (Figures 5a and b), which rules out a possible central clock dysfunction in the DEX-exposed mice. The transient increase in scaling exponent observed during forced synchronization by photic entrainment can be explained by a strong underlying rhythm, presumably diurnal. This effect was consistently detected in DEX-exposed mice at all ages, but in controls it reached significance only at the ages of 1 and 3 mo (Figure 5b). During steady entrainment, DEX-exposed mice displayed consistently higher scaling exponent than in free-running conditions, indicating that photic entrainment induces a more pronounced background rhythm in spontaneous activity in DEX-exposed mice. Taken together, these data confirm that the core alteration is the impairment in circadian entrainment.

### Reduced amplitude of oscillations in clock gene expression in primary skin fibroblasts derived from DEX-exposed mice

Skin fibroblasts express functional molecular clock machinery,^19, 20^ and the circadian oscillations in clock gene expression maintain to a large extent the features of circadian rhythms in the central clock.^21, 60^ The molecular clock machinery in the fibroblasts acts as a peripheral oscillator and is subject to entrainment by the central oscillator (located in the suprachiasmatic nucleus),^60^ similar to spontaneous activity.^61^ The possibility to synchronize self-sustained oscillations, and to reset the phase is preserved in cultured fibroblasts.^20, 22^ We therefore investigated the expression of clock genes in fibroblast isolated from controls and DEX-exposed mice aged 6 mo, the age when we documented the facilitated entrainment of spontaneous activity. After synchronization, the cells were harvested at different time points and the oscillations in *Bmal1* mRNA expression were investigated by cosinor rhythmometry.^38, 39^ Fibroblasts isolated from adult DEX-exposed mice displayed a smaller amplitude of oscillations in *Bmal1* mRNA expression (Figure 5c). This indicates that while the expression of *Bmal1* can be synchronized across the fibroblast cell population, the cross-synchronization dissipated faster in the fibroblasts derived from DEX-exposed mice, and is consistent with the facilitated circadian re-entrainment we observed in spontaneous activity.

### Circadian entrainment is not altered in a model of depression that is reversed by FLX

**Figure 6:**
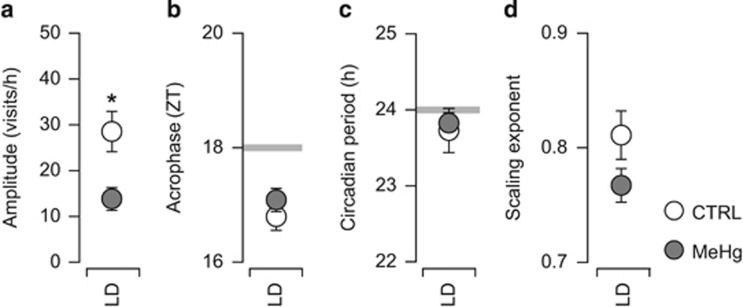
Circadian rhythmicity and entrainment in a model of depression induced by developmental exposure to MeHg. (**a**) Lower amplitude of circadian fluctuations in activity in MeHg-exposed mice. (**b**) Robust anticipation of phase change in MeHg-exposed mice, similar to controls (see Figure 3c for comparison). (**c**) The circadian period is about 24 h in both controls and MeHg-exposed mice, indicating robust circadian entrainment. (**d**) The scaling exponent indicates that spontaneous activity has fractal-like scale-invariant fluctuations and lacks exaggerated background rhythmicity, as seen in DEX-exposed mice (see Figure 5b for comparison). *N*=10 per group. *P<0.05, Student's *t*-test. LD, light–dark cycle; MeHg, methylmercury; ZT, zeitgeber time.

We reported earlier persistent depression-like behavior associated with impaired neurogenesis induced by developmental exposure to low levels of the food contaminant MeHg.^4, 16^ In contrast to the DEX-exposed mice, depression appeared at young age, and FLX treatment could restore neurogenesis and reverse the behavioral phenotype.^4, 15^ To evaluate the possible occurrence of alterations in circadian entrainment associated with MeHg-induced depression, we reanalyzed the available recordings of spontaneous activity under steady entrainment conditions (see Onishchenko *et al.*^16^). The amplitude of diurnal fluctuations in spontaneous activity was lower than in controls (Figure 6a), however, MeHg-exposed mice showed robust anticipation of phase change (acrophase occurring consistently before ZT 18; Figure 6b), maintained a circadian period close to 24 h (Figure 6c) and the scaling exponent was similar to controls (Figure 6d). Taken together, these findings indicate that, in contrast to DEX-exposed mice, circadian entrainment is not impaired in MeHg-exposed mice.

## Discussion

Here we show that an adverse prenatal milieu caused by excess GC induces alterations in circadian entrainment that precede the onset of FLX-resistant depression-like behavior. Impaired circadian entrainment was present already in mice 1 mo old and increased in severity with age. Young mice prenatally exposed to DEX exhibited rigid synchronization with the LD cycle and lacked the anticipation of phase change. By the age of 12 mo, circadian entrainment of spontaneous activity was lost and the depressive phenotype, not responding to FLX, became apparent.

Mice exposed to DEX displayed impaired hippocampal neurogenesis, which was not restored by FLX treatment. The decrease in neurogenesis may be due to epigenetic reprogramming, as we have previously reported in primary neural stem cell cultures exposed to DEX.^14^ Relevant to the present study, the effects on proliferation, neuronal differentiation as well as the expression of senescence markers in neural stem cell cultures was heritable, persisting long after the actual exposure to DEX had ceased.^14^

The absence of circadian oscillations in hippocampal clock gene expression may also contribute to the decrease in neurogenesis in DEX-exposed mice. In fact, the proliferation and differentiation of neuronal progenitors in the subgranular zone is controlled by clock genes^30, 31^ and the circadian oscillations in their expression is influenced by GC.^54, 55^ Rhythmic GC secretion seems to be required also for FLX to restore neurogenesis.^49^ Therefore, the arrhythmic GC secretion and the associated alterations in clock gene expression may explain not only the impaired neurogenesis, but also the lack of effect of FLX in DEX-exposed mice. Notably, the alterations in hippocampal clock gene expression were detected simultaneously with the first consistent changes in circadian rhythms in spontaneous activity, long before the onset of depression.

Circadian disturbances are often found in subjects with depression, and it has been hypothesized that the core alteration is the lack of synchronization of internal oscillators with environmental stimuli (reviewed in the studies by Edgar *et al.*^17^ and Landgraf *et al.*^62^). In experimental models, prolonged deprivation of photic entrainment has been shown to induce depression- and anxiety-like behavior.^63, 64^ Here we report that progressive alterations in circadian entrainment precede the onset of FLX-resistant depression-like behavior. In DEX-exposed mice aged 12 mo, the photic stimuli have a strong effect on spontaneous activity, but appear not to be integrated as diurnal entrainment at organismal level. A possible cause could be the failure of circadian function in the hypothalamic–pituitary–adrenal axis, which subsequently leads to depression-like behavior. To investigate the relevance of circadian entrainment for antidepressant treatment response, we reanalyzed the spontaneous activity in a model of depression induced by prenatal exposure to MeHg. As we reported earlier, MeHg induces persistent depression-like behavior that can be reversed by FLX.^4, 15^ In contrast with the DEX-exposed mice, depression in MeHg-exposed mice was not accompanied by alteration in circadian entrainment. Therefore, altered circadian entrainment appears to be specific for FLX-resistant depression models.

Skin fibroblasts express functional molecular clock machinery,^19, 20^ and circadian oscillations in clock gene expression in cultured fibroblasts have been shown to mirror the features of circadian rhythms in healthy subjects as well as in psychiatric patients.^21, 22, 23^ Here, we show that the amplitude of oscillations in *Bmal1* expression in cultured fibroblasts is decreased in DEX-exposed mice, which is consistent with the alterations in circadian entrainment we observed in their spontaneous activity. Relevantly, these alterations were detected before the onset of depression-like behavior, but simultaneously with the altered circadian rhythmicity during steady entrainment.

Our findings, if implemented in a clinical setting, may bring new hope to patients with major depressive disorder and to the clinicians responsible for their treatment. A bioassay, based on the present results, could avoid months of trial and error to find the drug to which a patient responds. Moreover, it could allow early identification of subjects at risk for depression by targeted monitoring aiming at prompt intervention.

In conclusion, we have shown that alterations in circadian entrainment precede the onset of FLX-resistant depression-like behavior. Therefore, we propose that the analysis of circadian entrainment has a potential prognostic value in predicting the onset of depression, and possibly the response to FLX and similar antidepressant treatments.

## Notes

The data presented here form the basis of a patent application (SS and SC). The remaining authors declare no conflict of interest.
