# Supplementary material for: Alterations in circadian entrainment precede the onset of depression-like behavior that does not respond to fluoxetine
Source: Transl Psychiatry. 2015 Jul 14;5(7):e603–. doi: 10.1038/tp.2015.94 (PMC5068723; doi:10.1038/tp.2015.94)
Supplement: Supplementary Information [file tp201594x1.doc]

**Supplementary Information**

Supplementary Materials and Methods

Supplementary Fig. 1. Growth retardation induced by prenatal exposure to DEX.

Supplementary Fig. 2. DEX-exposure induces hyperactivity and impaired social behavior.

Supplementary Fig. 3. Tail-suspension test at the age of 12 mo.

Supplementary Fig. 4. GR expression in the hippocampus.

Supplementary Table 1. Antibodies used for immunohistochemical analyses.

Supplementary Table 2. Primer sequences for qPCR analyses.

**Supplementary Materials and Methods**

*Open field test*

For the open field test, the mice were moved to the experimental room at least 30 min before the experiment. The animals were individually placed in fresh cages identical to the homecage, and the locomotor activity was videotaped for 60 min (6 10 min bins) and analyzed online using a TopScan™ system (CleverSys Inc., Reston, VA, USA). All animals in each cage were tested simultaneously in cages widely spread and visually shielded from one another.

*Social recognition test*

The social recognition test was performed as described in ^62^. The experimental mice were housed in individual cages (small size; referred as “home cages” in the description of this test) in order to induce territoriality in the test animals and to avoid activity-stimulating effect of a novel cage. The animals were transferred to the testing room and allowed to acclimatize overnight in similar conditions as in the holding facility. We used clear Plexiglas cylinders (7 cm in diameter, 12 cm high) and 36 holes (4 mm diameter) at the lower part of the walls to allow the passage of olfactory cues, but prevent direct interaction between the animals. Before the 1^st^ trial, the test mice were allowed to explore freely the empty cylinder in the home cage for 10 min. A mouse from the same strain, sex and age (“intruder”) was then introduced inside the cylinder before placing the cylinder in the home cage. The intruder mice were habituated with the procedure for 3 days prior to the testing day in order to minimize the stress. The mice were allowed to freely approach and explore the intruder for 5 min in 5 trials with 15 min between-trial interval. The same intruder was introduced 4 times, and the 5^th^ trial involved a new intruder. The test mice and their respective homecage were left undisturbed in the room for the entire duration of the testing session. The position of all the cylinders introduced into the mouse home cage was kept constant throughout the experiment. The behavior was videotaped with surveillance cameras placed 75cm above the cage, and the captured images were combined in a single video and stored for offline analysis. The behavior was scored manually by the same investigator. Social interaction was recorded when the test animal approached and sniffed the cylinder from a distance of less than 2cm for at least 2s. The number of approaches and the total duration was recorded and used for further analyses.

*Tail suspension test*

The test was performed as described by Steru et al.^63^, with minor modifications. The mice were allowed to acclimatize in the testing room for at least 30 min before the experiment. The mice were suspended by the tail 15 cm above the table (using adhesive tape placed approx. 2 cm from the base of the tail). Soft padding was provided in order to prevent injuries in case the animal escaped or fell down because of tape failure. After 6 min, the animal were released and placed in the homecage. The experiment was videotaped and the analysis was performed offline by a rater blind to the experimental conditions. The immobility time was estimated by summing the duration of immobility bouts longer than 3s. Animals that displayed persistent climbing on the tail (total duration exceeding 72s), or escaped were excluded from analysis.

*Immunohistochemistry*

Control and DEX-exposed 12 mo mice were killed by an overdose of anesthetic (sodium pentobarbital, 150mg/kg). The brain was fixed by intracardial perfusion with ice-cold paraformaldehyde (4% in PBS, 100 ml/animal), then postfixed overnight at 4°C in paraformaldehyde before cryoprotection in 10% buffered sucrose (overnight at 4°C) and stored at -80°C until processing. The brains were cut in sagittal sections (20 µm thick) with a cryostat (Leica CM3050). Equally spaced series (200 µm between consecutive slices) were collected starting from the first occurrence of the hippocampal structure until the dorsal hippocampal commissure (lateral 3.5 – 0 mm in stereotaxic coordinates ^27^) and stored at -80°C until processing. The slides were air-dried for 30 min at room temperature, then rehydrated for 10 min in PBS. Before the application of the primary antibody, the unspecific labeling was blocked by incubation for 2 h with normal serum of the species in which the secondary antibody was raised (in 0.3% Triton-X in PBS, 50 µl/slice). The slices were then incubated with the primary antibodies at 4°C (see Supplementary Table I for incubation time and dilution for each antibody). After washing, the tissue slices were incubated with the secondary antibody for 2h at room temperature. The excess secondary antibody was removed by repeated washing with PBS, then counterstained using a fluorescent nuclear dye (DAPI, Sigma-Aldrich, Germany) before mounting the slides with fluorescent mounting medium (Dako, Golstrup, Denmark). The specificity of the staining was verified by using the same protocol, except omitting the primary antibody from the cocktail for the first incubation.

*RNA extraction, cDNA synthesis, and PCR amplification*

For hippocampal samples, the RNA was extracted using the TRI™ Reagent (Sigma-Aldrich, Germany) following the protocol provided by the manufacturer. Total mRNA was extracted from cultured fibroblasts using PeqGOLD™ Total RNA kit (peQlab, Erlangen, Germany) as instructed by the manufacturer. The quality of the extracted RNA was checked using a NanoDrop™ 1000 UV spectrophotometer (Thermo Scientific, Wilmington, DE, USA). cDNA was prepared using a Superscript II first strand cDNA synthesis kit (Invitrogen Inc., Carlsbad, CA, USA) starting from 2 µg total RNA and 0.5 µg oligo-dT primers. PCR amplification reactions were performed using 0.5 µl cDNA and SYBR® Green PCR Master Mix (Applied Biosystems, Life Technologies Corp., Warrington, UK) and 0.2 µM forward and reverse primers (total reaction volume adjusted to 12.5 µl with DNAse- and RNAse-free water) in a 7500 Fast Real-Time PCR System (Applied Biosystems) running 7500 software version 2.05. The negative control reactions contained water instead of cDNA template. The primer sequences are listed in Supplementary Table 1. The PCR cyclic amplification was performed for 1 min at 60°C for 45 cycles. The specificity of the amplification was checked by inspection of the melting curve and by electrophoresis in 2% agarose gel. The relative expression regulation was calculated as with GAPDH as housekeeping gene. All amplification reactions were run in technical duplicates, and all experiments were repeated 3 times.


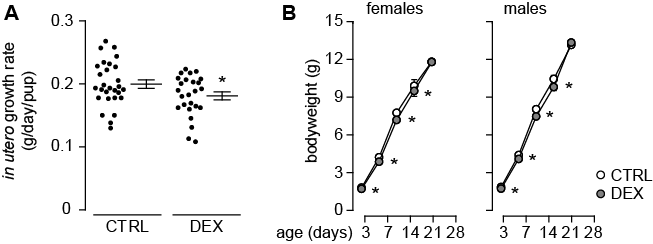
**Supplementary Figures**

**Supplementary Fig. 1.** Growth retardation induced by prenatal exposure to DEX. (A) DEX treatment of the pregnant female mice induces a decrease in the daily growth rate. (B) Both male and female DEX-exposed pups have lower body weight than controls up to weaning (PND 21).

**
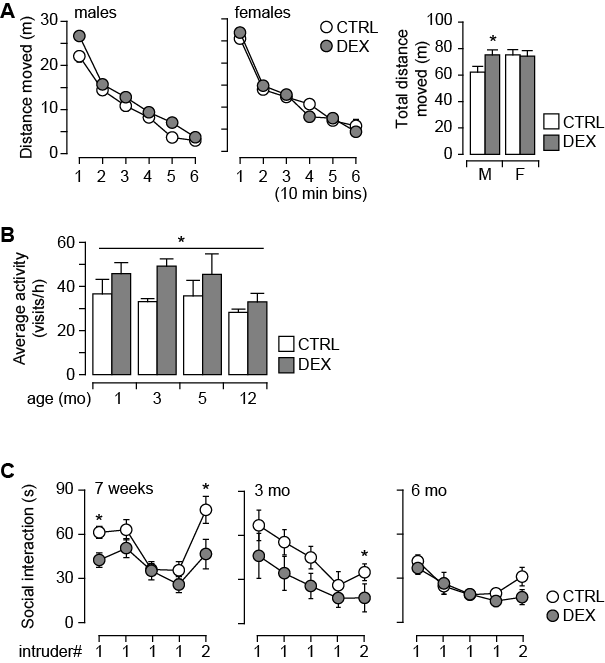
**

**Supplementary Fig. 2.** DEX-exposure induces hyperactivity and impaired social behavior. (A) Developmental exposure to DEX induces hyperactivity (increased total activity) in the open field at 5-6 weeks only in male pups. * p<0.05, factorial ANOVA followed by unequal N HSD post-hoc test. (B) The hyperactivity in DEX-exposed male mice can be documented also in the homecage as increased average hourly activity in 12:12h LD cycle. The effect is consistently found from 1 to 12 mo. * p<0.05, factorial ANOVA, followed by contrast analysis. (C) Impaired social behavior shown as decreased interest in a new conspecific. The effect is present only in male offspring, and is consistent across ages. *p<0.05, repeated measures ANOVA followed by unequal N HSD post-hoc test. N=7-8/group

**
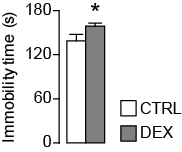
**

**Supplementary Fig. 3.** Tail suspension test at 12 mo. DEX-exposed mice show increased immobility time, in line with the findings in forced swim test. CTRL N=8; DEX: N=10;* p<0.05, student’s t-test.

**
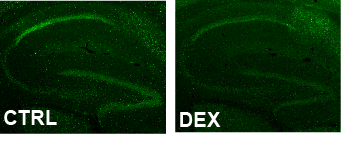
**

**Supplementary Fig. 4.** GR expression in the hippocampus (immunohistochemical staining; see main text for details and quantification). The intensity of the fluorescence signal is decreased throughout the hippocampal formation in DEX-exposed mice.

**Supplementary Tables**

**Supplementary Table 1 Antibodies used for immunohistochemical analyses**

| antigen | manufacturer | Cat No. | dilution | incubation time |
| --- | --- | --- | --- | --- |
| GR | Santa Cruz Biotechnology | GR H-300 | 1:500 | 72 h |
| DCX | Millipore Inc | AB2253 | 1:1000 | overnight |

**Supplementary Table 2 Primer sequences for qPCR analyses**

| primer | sequence |
| --- | --- |
| GAPDH | fw CAAGGCCGAGAATGGGAAG  rv GGCCTCACCCCATTTGATGT |
| Bmal1 | fw AACCTTCCCGCAGCTAACAG  rv AGTCCTCTTTGGGCCACCTT |
| Per 1 | fw CCAGATTGGTGGAGGTTACTGAG  rv GCGAGAGTCTTCTTGGAGCAGTA |
| Rev-Erb α | fw GGAACGGACCGTCACCTTT  rv TCCCCTGCTCCCATTGAGT |
